# 

**Published:** 1927

**Authors:** 


### The Medical Library of the University of Bristol,

WITH WHICH IS INCORPORATED

**The Library of the Bristol Medico-Chirurgical Society.**

*The following donations have been received since the publication of the List in September,* 1927.

December, 1927.

| | | |
|---|---|---|
| College of Physicians and Surgeons, Columbia University (1) .. .. .. .. | 1 | volume |
| Dr. D. S. Davies (2) .. .. .. .. | 1 | ,, |
| Founder and Director, Wellcome Historical Medical Museum (3) .. .. .. | 1 | ,, |
| Henry Phipps Institute (4) .. .. .. | 1 | ,, |
| Lister Centenary Committee (5) .. .. | 1 | ,, |
| London School of Tropical Medicine and Hygiene (6) .. .. .. .. .. | 1 | ,, |
| Dr. J. E. Mackenty (7) .. .. .. | 1 | ,, |
| Mayo Clinic (8) .. .. .. .. .. | 1 | ,, |
| Michell Clarke Memorial Fund (9) .. .. | 3 | volumes |
| Munro Smith Memorial Fund (10) .. .. | 6 | ,, |
| Registrar-General (11) .. .. .. .. | 1 | volume |
| Rockefeller Foundation (12) .. .. .. | 1 | ,, |
| Saito Gratitude Foundation (13) .. .. | 1 | ,, |
| Mr. A. Rendle Short (14) .. .. .. | 1 | ,, |
| Surgeon-General of the U.S. Army (15) .. | 3 | volumes |
| United Fruit Co. (16) .. .. .. .. | 1 | volume |
| University of Liège (17) .. .. .. | 1 | ,, |
| Col. H. S. Wood (18) .. .. .. .. | 7 | volumes |
| Mr. A. J. M. Wright (19) .. .. .. | 1 | volume |

Unbound periodicals have also been received from Col. P. Bush, Dr. Carey Coombs and Professor E. W. Hey Groves.

### THE ONE HUNDRED AND TWENTY-SECOND LIST OF BOOKS.

The figures in round brackets refer to the figures after the names of the donors. The books to which no such figures are attached have either been bought from the Library Fund or received through the *Journal.*

**Bailey, H.** .. .. .. *Physical Signs in Clinical Surgery* .. .. .. 1927
**Buchanan** .. .. .. *Manual of Anatomy.* 2 Copies .. 5th Ed. (10) 1925
**Comrie, J. D.** .. .. *History of Scottish Medicine to* 1860 .. .. (3) 1927
*Concise Oxford Dictionary of Current English* .. .. .. .. .. .. .. 1926
**Coope, R.** .. .. .. *Diagnosis of Pancreatic Disease* .. .. .. .. 1927
**Crow, D. A.** .. .. *Ear, Nose and Throat in General Practice* .. 1927
**Cuff, H. E., and Pugh, W. T. G.** *Practical Nursing* .. .. .. 7th Ed. 1927
**Dautrebande, L.** .. *Troubles Circulatoires dans l'asystolie* .. (17) 1926
**Evans, C. L.** .. .. *Recent Advances in Physiology* .. 2nd Ed. (14) 1926
**Ewart** .. .. .. .. *MS. Notes on Lectures on Zoology.* 2 Vols. (18) —
**Feldman, W. M.** .. *Ante-Natal and Post-Natal Physiology* .. (10) 1920
**Gray, H.** .. .. .. *Anatomy* .. .. .. .. .. .. .. 23rd Ed. (10) 1926
**Hehir, Sir P.** .. .. *Malaria in India* .. .. .. .. .. .. .. .. 1927
**Hovorka, O. von, and Kronfeld, A.** *Vergleichende Volksmedizin.* 2 Vols. 1908–1909
**Jackson, J. H.** .. .. *Neurological Fragments* .. .. .. .. .. (10) 1925
**Jacobi** .. .. .. .. *Atlas of Dermochromes.* By H. MacCormac. 2 Vols. .. .. .. .. .. .. .. 4th Ed. (9) 1926
**Mackenty, J. E.** .. *Cancer of the Larynx* .. .. .. .. (7) [1927]
**MacLagan** .. .. .. *MS. Notes on Lectures on Medical Jurisprudence* (18) —
**Manson-Bahr, H., and Alcock, A.** *Life and Work of Sir Patrick Manson* 1927
*Medical Department of United States Army in World War.* Vol. II., XI., I. (15) 1927
*Modern Technique in Treatment.* Vol. III. .. .. .. .. .. .. .. .. 1927
*Nouveau Traité de Médécine. Fasc.* XXI. .. .. .. .. .. .. (9) 1927
**Porter, C.** .. .. .. *Elements of Hygiene and Public Health.* 2nd Ed. 1926
**Portmann, G., and Retrouvey, H.** *Cancer du Nez* .. .. .. .. (19) 1927
**Ray, M. B.** .. .. *Rheumatic Diseases* .. .. .. .. .. .. .. .. 1927
**Rudolf, G. de M.** .. *Therapeutic Malaria* .. .. .. .. .. .. .. 1927
**Schwanzy** .. .. .. *Diseases of the Eye.* Ed. Werner .. .. 13th Ed. 1925
**Shaw, D. M.** .. .. *Dental Prosthetic Mechanics* .. .. .. .. .. .. 1927
**Sykes, W. S.** .. .. *Manual of General Medical Practice* .. .. .. 1927
**Turner, A. Logan** .. *Diseases of the Nose, Throat and Ear* .. 2nd Ed. 1927
**Turner, A. Logan** [Ed.] *Joseph, Baron Lister,* 1827–1927 .. .. .. (5) 1927
**Wells, H. G.** .. .. *Chemical Pathology* .. .. .. .. .. .. .. (10) 1925

### TRANSACTIONS, REPORTS, JOURNALS, ETC.

Acta Pathologica et Microbiologica Scandinavica. New journal. Vol. 1 (1924). Vol. 3 (1926) and in continuation.
Annual Report of the Medical Officer of Health, Bristol .. .. .. (2) 1926
Collected Papers of the Mayo Clinic. Vol. 18 .. .. .. .. .. .. (8) 1926
Henry Phipps Institute. 19th Report .. .. .. .. .. .. .. .. (4) 1927
Index Catalogue Surgeon-General Office United States Army. 3rd S. Vol. 6 .. .. .. .. .. .. .. .. .. .. .. .. .. .. .. .. (15) 1926
London School of Tropical Medicine. Collected Essays. Vol. 3 .. (6) 1926–27
Progressive Medicine. Vol. 3. 1927 .. .. .. .. .. .. .. .. .. 1927
Quarterly Cumulative Index Medicus. Vol. 1. 1927 .. .. .. .. .. 1927
Registrar-General's Decennial Supplement. England and Wales. Pt. I. .. 1927
Registrar-General's Statistical Review. England and Wales. Tables. Pt. I. Medical for 1926 (11).

Registrar-General's Statistical Review. England and Wales. Tables. Pt. II. Civil for 1926. (11).
Rockefeller Foundation. Annual Report .. .. .. .. .. .. .. (12) 1926
St. Thomas's Hospital Reports. Vol. 49 (1925) .. .. .. .. .. .. .. 1927
Saito Gratitude Foundation. Monographs. No. 2 .. .. .. .. (13) 1927
Studies from Department of Pathology, College of Physicians and Surgeons, Columbia University. Vol. 20 (1) .. .. .. .. .. .. .. .. 1925–1927
Surgical Clinics of North America. Vol. 7. Nos. 3 and 4 .. .. .. 1927
Transactions of College of Physicians, Philadelphia. Vol. 48 .. .. .. 1926
United Fruit Co., Medical Department. 15th Annual Report .. .. (36) 1926

### Local Medical Notes.

**Royal College of Surgeons of England.**—The Thomas Vicary Lecture for 1927 was delivered on November 3rd by Dr. George Parker, Consulting Physician to the Bristol General Hospital, whose subject was "The Early Development of Hospitals."

**Bristol General Hospital.**—The following appointments have recently been made :—Consulting Physician : Dr. J. O. Symes. Physician : Dr. Carey Coombs. Assistant Physician : Dr. J. Anthony Birrell. Assistant Surgeon in the Ear, Nose and Throat Department : Mr. Gordon R. Scarff. Assistant Pathologist : Dr. H. Rogers.

**Bristol Royal Infirmary.**—C. C. Ross, M.D. Manitoba, F.R.C.S. (Edin.), has been appointed Senior Resident Officer.

### EXAMINATION RESULTS.

**University of Bristol.**—Students of the University have recently passed the following examinations :—

M.B., Ch.B.—*Supplementary First Examination. Pass :* R. N. O. Burns, R. G. C. G. Carlson, J. D. Hughes, J. J. Kempton, J. Ridgway. *In Physics and Biology only :* J. F. H. Eagles.

M.B., Ch.B.—*Final Examination (Part I.), including Forensic Medicine and Toxicology. Pass :* R. A. S. Cory, A. A. Dowling, G. R. Fells (with distinction in Forensic Medicine and Toxicology), A. C. Fisher, N. D. Gerrish, R. J. Krogh, K. H. Pridie (with distinction in Materia Medica, Pharmacy, Pharmacology and Therapeutics), H. D. Pyke, N. C. Tanner (with distinction in Materia Medica, Pharmacy, Pharmacology and Therapeutics), L. E. Vine, G. J. West. *Part I. only. Pass :* Jean Armstrong, T. Evans, A. C. Price.

M.B., Ch.B.—*Final Examination (Part II.).—Completing Examination. Pass :* G. D. J. Ball, T. A. Brand (with distinction in Public Health), J. E. Martin, Dorothy M. H. Tripp, A. R. Williams. *In Group I. only :* D. E. C. Andrew, T. L. Cleave, A. J. M. Grimston, Phoebe C. Vine.

SUPPLEMENTARY PRELIMINARY SCIENCE EXAMINATION FOR DENTAL STUDENTS.—*Pass :* Helena C. L. Blinkworth. *In Physics (completing Examination) :* H. L. Saunders.

B.D.S.—*Supplementary First Examination. Pass :* M. J. Ryan.

L.D.S.—*Second Professional Examination. Pass : In Dental Materia Medica (completing Examination) :* L. W. G. Jones. *In Dental Metallurgy and Dental Mechanics only :* Barbara J. Shapland. *In Dental Materia Medica only :* A. N. Moon, T. A. Satchwell, C. G. Westlake.

L.D.S.—*Final Professional Examination. Pass :* E. C. Browne, A. L. Gilchrist.

**University of London.**

M.D.—J. A. James, B.S.

**Conjoint Board.**

M.R.C.S., L.R.C.P.—T. I. Hughes.

L.D.S., R.C.S.—*First Professional Examination. Part I. :* J. D. Sellers, A. H. V. Williams. *Part II. :* M. E. Minton, J. P. Price, J. D. Sellers. *Part III. (a) :* A. H. V. Williams, G. H. Dakers, Q. A. Davies, T. A. A. Eaves, W. F. G. Harvey, J. J. Hinton, D. R. Jones, J. K. Noot, H. J. Phillys, K. V. M. Taylor-Milton. *Part III. (b) :* F. G. Lilley, L. Symonds.

**The Annual Medical Dinner** of the Bristol Medical School was held on Thursday, December 8th, at the Berkeley Café. A large gathering of past and present students and members of the staff assembled under the presidency of Professor J. A. Nixon, C.M.G., F.R.C.P., the guest of the evening being Col. T. Sinclair, C.B., M.P. for the University of Belfast.

After the loyal toast the President proposed the health of Col. Sinclair, introducing him as having been until recently Professor of Surgery (Queen's University, Belfast), late Consulting Surgeon in Egypt and to the Fourth Army in France, and now the first Member of Parliament to come amongst them as their honoured guest.

Col. Sinclair, in responding, proposed "The Bristol Medical School," and Col. J. Paul Bush, C.M.G., C.B.E., and Mr. R. H. Moore replied appropriately for the School.

Dr. S. V. Stock's toast of "The President" and Professor Nixon's reply concluded a most enjoyable evening.

### Index.

Acute Suffocative Œdema of the Lungs. —J. A. Birrell, 239.
Addisonian Anæmia, The Central Nervous System in.—G. Hadfield, 21.
Anæsthesia.—A. L. Flemming, 1.
Angina Pectoiis following Sepsis, Two Cases of.—Newman Neild, 263.
Annual Service for the Medical Profession, The, 276.
Arrowsmith-Brown, J. A., Sheriff of Bristol, 274.

Baker, Dr. Lily, Complimentary Dinner to, 58.
Birrell, J. A.—Acute Suffocative Œdema of the Lungs, 239.
Blachford, J. V.—The recommendations of the Royal Commission on Lunacy and Mental Disorder, 15.
Books, Reviews of—
- Advice to the Expectant Mother.—F. J. Browne, 49.
- Alcock, A. and Manson-Bahr, P. H.—The Life and Work of Sir Patrick Manson, 218.
- Atlas of the History of Medicine.—J. G. de Lint, 52.
- Bailey, H.—Physical Signs in Clinical Surgery, 271.
- Bayly, H. W.—Venereal Disease, 218.
- Belfrage, S. H.—What 's Best to Eat, 209.
- Bernhard, O.—Light Treatment in Surgery, 134.
- Bone, Inflammatory and Toxic Diseases of.—R. L. Knaggs, 53.
- Bousfield, P.—Functional Nervous Diseases, 50.
- Browne, F. J.—Advice to the Expectant Mother.—49.
- Bruce, J. M. and Dilling, W. J.—Materia Medica and Therapeutics, 132.
- Burke, E. T.—Treatment of Venereal Disease, 217.
- Cameron, S. J. and Hewitt, J.—Difficult Labour, 129.
- Chapman, C. W.—The Heart and its Diseases, 216.
- Chronic Intestinal Stasis.—A. C. Jordan, 216.
- Chronic Rheumatic Diseases.—F. G. Thomson and R. G. Gordon, 51.

Books, Reviews of (continued)—
- Cochrane, W. A. — Orthopædic Surgery, 270.
- Coope, R.—The Diagnosis of Pancreatic Disease, 27.
- Cotton, Wm.—Perspective in relation to X-ray Interpretation, 271.
- Cowell, E. M.—Hernia and Hernioplasty, 212.
- Crow, D. A.—The Ear, Nose and Throat in General Practice, 269.
- Cystoscopy.—J. B. Macalpine, 216.
- Dally, J. F. H.—High Blood Pressure, 137.
- De Lint, J. G.—Atlas of the History of Medicine, 52.
- Dental Anæsthesia.—G. F. R. Smith, 55.
- Dental Materia Medica.—P. H. Marsden, 54.
- Dental Prosthetics.—J. D. Logan, 136.
- Diabetes Mellitus, The Pathology and Treatment of.—G. Graham, 137.
- Diagnosis of Pancreatic Disease, The —R. Coope, 272.
- Difficult Labour.—S. J. Cameron and J. Hewitt, 129.
- Dilling, W. J. and Bruce, J. M.—Materia Medica and Therapeutics. 132.
- Diseases of the Nose, Throat and Ear. —A. Logan Turner, ed., 269
- Diseases of the Stomach, A Handbook of.—S. Wyard, 217.
- Ear, Nose and Throat in General Practice, The.—D. A. Crow, 269.
- Eason, J.—Exophthalmic Goitre, 215.
- Employment for the Tuberculous.—W. Bolton Tomson, 49.
- Epidemic Diseases of the Central Nervous System.—A .S. MacNalty, 213.
- Exophthalmic Goitre.—J. Eason, 215.
- Fifield, L. R.—Infections of the Hand, 129.
- Fremantle, F. E.—The Health of the Nation, 267.
- Functional Nervous Diseases.—P. Bousfield, 50.
- Gastro-Duodenal Ulceration, The Surgery of.—C. A. Pannett, 53.
- Gordon, R. G. and Thomson, F. G.—Chronic Rheumatic Diseases, 51,

Books, Reviews of (continued)—
Graham, G.—Pathology and Treatment of Diabetes Mellitus, 137.
Hair and your Health, Your.—O. L. Levin, 52.
Handbook of Midwifery and Gynæcology.—W. E. T. Haultain, 55.
Handbook for Queen's Nurses, 49.
Haultain, W. F. T.—Handbook of Midwifery and Gynæcology, 55.
Health of the Child of School Age, The.—Sir T. Oliver, 214.
Heart and its Diseases, The.—C. W. Chapman, 216.
Heart, Diseases of the.—F. W. Price, 211.
Heart and Lungs, Diseases of, for Nurses.—A. I. G. McLaughlin, 133.
Hehir, Sir P.—Malaria in India, 272.
Heliotherapy.—A. Rollier, 130.
Hernia and Hernioplasty.—E. M. Cowell, 212.
Hewat's Examination of the Urine.—G. L. M. Smith, 131.
Hewitt, J. and Cameron, S. J.—Difficult Labour, 129.
High Blood Pressure.—J. F. Halls Dally, 137.
Hurry, J. B.—Imhotep, 210.
Hutchison, R.—The Elements of Medical Treatment, 210.
Imhotep.—J. B. Hurry, 210.
Infections of the Hand.—L. R. Fifield, 129.
Introduction to the Law and Tradition of Medical Practice, An.—Wm. Sanderson and E. B. A. Rayner, 272.
Irwin, W. K.—Urinary Surgery, 220.
Jack, W. R.—Wheeler's Handbook of Medicine, 130.
Jordan, A. C.—Chronic Intestinal Stasis, 216.
Knaggs, R. L.—The Inflammatory and Toxic Diseases of Bone, 53.
Lees, D.—Diagnosis and Treatment of Venereal Diseases, 211.
Lenk, R.—X-ray Therapy, 136.
Levin, O. L.—Your Hair and Your Health, 52.
Light Treatment in Surgery.—O. Bernhard, 134.
Logan, J. D.—Dental Prosthetics, 136.
Love, R. J. M.—A Shorter Surgery, 132.
Macalpine, J. B.—Cystoscopy, 216.
McLaughlin, A. I. G.—Diseases of the Heart and Lungs, for Nurses, 133.
MacNalty, A. S.—Epidemic Diseases of the Central Nervous System, 213.
Malaria in India.—Sir P. Hehir, 272.

Books, Reviews of (continued)—
Manson, Sir P., The Life and Work of.—P. H. Manson-Bahr and A. Alcock, 218.
Manson-Bahr, P. H. and Alcock, A.—The Life and Work of Sir P. Manson 218.
Manual of General Medical Practice. A.—W. S. Sykes, 271.
Marsden, P. H.—Dental Materia Medica, 54.
Materia Medica and Therapeutics.—J. M. Bruce and W. J. Dilling, 132.
Medical Treatment, Elements of.—R. Hutchison, 210.
Medicine, Manual of.—A. S. Woodwark, 212.
Medicine, The Study of.—A. Miles, 209.
Miles, A.—The Study of Medicine, 209.
Miller, E.—Types of Mind and Body, 133.
Mind and Body, Types of.—E. Miller, 133.
Monrad - Krohn, G. H.— Clinical Examination of the Nervous System, 135.
Nervous System, Clinical Examination of.—G. H. Monrad-Krohn, 135.
Nose and Throat, Diseases of.—Sir St. C. Thomson, 55.
Oliver, Sir. T—The Health of the Child of School Age, 214.
Orthopædic Surgery.—W. A. Cochrane, 270.
Pannett, C. A.—The Surgery of Gastro-Duodenal Ulceration, 53.
Perspective in Relation to X-ray Interpretation.—Wm. Cotton, 271.
Physical Signs in Clinical Surgery.—H. Bailey, 271.
Price, F. W.—Diseases of the Heart, 211.
Ray, M. B.—Rheumatic Diseases, 270.
Rayner, E. B. A. and Sanderson, Wm.—An Introduction to the Law and Tradition of Medical Practice, 272.
Redding, J. Magnus.—X-ray Diagnosis, 51.
Rheumatic Diseases.—M. B. Ray, 270.
Rollier, A.—Heliotherapy, 130.
Rudolf, G. de M.—Therapeutic Malaria, 219.
Sanderson, W. and Rayner, E. B. A.—An Introduction to the Law and Tradition of Medical Practice, 272.
Smith, G. F. R.—Dental Anæsthesia, 55.

BOOKS, REVIEWS OF (continued)—
Smith, G. L. M.—Hewat's Examination of the Urine, 131.
Surgery, Fundamentals in the Art of. —J. H. Watson, 134.
Surgery, A Shorter.—R. J. M. Love, 132.
Surgical Applied Anatomy.—Sir F. Treves, 131.
Sykes, W. S.—A Manual of General Medical Practice, 271.
Therapeutic Malaria.—G. de M. Rudolf, 219.
Thomson, F. G. and Gordon, R. G.—Chronic Rheumatic Diseases, 51.
Thomson, Sir St. C.—Diseases of the Nose and Throat, 55.
Tomson, W. Bolton.—Notes, etc., on Finding Employment for the Tuberculous, 49.
Treves, Sir F.—Surgical Applied Anatomy, 131.
Turner, A. Logan.—Diseases of the Nose, Throat and Ear, 269.
Urinary Surgery.—W. K. Irwin, 220.
Venereal Disease.—H. W. Bayly, 218.
Venereal Disease, Treatment of in General Practice.—E. T. Burke, 217.
Venereal Diseases, Diagnosis and Treatment of.—D. Lees, 211.
Watson, J. H.—Fundamentals in the Art of Surgery, 134.
What 's Best to Eat.—S. H. Belfrage, 209.
Wheeler's Handbook of Medicine.—W. R. Jack, 130.
Woodwark, A. S.—Manual of Medicine, 212.
Wyard, S.—A Handbook of Diseases of the Stomach, 217.
X - Ray Diagnosis.— J. Magnus Redding, 51.
X-ray Therapy.—R. Lenk, 136.
Bright, Richard. — Commemoration, 221.
Bristol Medical School and its Association with the University College.—F. R. Cross, 74.
Buckmaster, G. A.—Some Considerations on the Cells and Liquids of the Body, 154.

Cancer Arising in Tissues within the Duodenal Loop.—Helen B. Murgoci, 195.
Cancer, The treatment of.—A. T. Todd, 144.
Cells and Liquids of the Body.—G. A. Buckmaster, 154.
Central Nervous System in Addisonian Anæmia, The.—G. Hadfield, 21.
Colston Research Society, The. —Annual Dinner, 139.
Coombs, C. F.—Coronary Obstruction, 249.
Coronary Obstruction.— Carey F. Coombs, 249.
Coronary Occlusion, The Pathology of. —G. Hadfield, 257.
Cross, F. Richardson.—Early Medical Teaching in Bristol, 73; Lister—Reminiscences of Fifty Years Ago, 113.

DISCUSSIONS :
The Recommendations of the Royal Commission on Lunacy, etc.—J. V. Blachford. Discussed by A. L. Flemming, 59; E. B. White, 59; J. R. P. Phillips, 59; J. A. Nixon, 60.
Prophylaxis of Ophthalmia Neonatorum.—A. E. Iles. Discussed by A. L. Flemming, 141; E. R. Chambers, 142; C. H. Walker, 142; R. S. S. Statham, 142; D. A. Alexander, 142.
Vertigo, The Significance of.—E. Watson-Williams. Discussed by A. J. Wright, 143; P. Watson-Williams, 143; H. H. Carleton, 143; E. R. Chambers, 143; D. A. Alexander, 144.
Cancer, The Treatment of.—A. T. Todd. Discussed by E. Watson-Williams, 145; A. R. Short, 146; R. S. S. Statham, 146; A. W. Adams, 146; W. A. Jackman, 147; F. H. Edgeworth, 147; A. L. Flemming, 147; G. B. Bush, 147; D. A. Alexander, 147.
Dysmenorrhœa.— R. S. Statham. Discussed by A. L. Flemming, 223; R. C. Leonard, 223; R. Burges, 223; R. G. Gordon, 223; R. S. Carey, 223; A. E. J. Lister, 223; G. T. Myles, 224; J. L. Firth, 224; W. A. Jackman, 224; J. H. C. Eglinton, 224.
Interlobar Empyema.— Newman Neild. Discussed by W. K Wills, 280; C. F. Walters, 280; C. A. Moore, 280; H. H. Carleton, 280; J. R. Charles, 281.
Dysmenorrhœa, Some Clinical Aspects of.—R. S. S, Statham, 187.

Endometriomata, The.—A. D. Fraser, 113.

Flemming, A. L.—Anæsthesia, 1.
Fraser, A. D.—Endometriomata, 113.

Groves, E. W. H.—Hospitals of Madrid, 229.

Hadfield, G.—The Central Nervous System in Addisonian Anæmia, 21; The Pathology of Coronary Occlusion, 257.
Hadfield, G. and White, E. B.—Observations of Pellagra, 31.
Health of the Nation, The.—A Review. —The Editor, 267.
Henry Herbert Wills Physics Laboratory, The, Opening of, 275.
Hospitals of Madrid, The.—E. W. Hey Groves, 229.

Iles, A. E.—The Prophylaxis of Ophthalmia Neonatorum, 125.

Lister—Reminiscences of Fifty Years Ago.—F. R. Cross, 113.
Lister, Lord—Centenary, 138.
Local Medical Notes, 64, 151, 228, 283.
Long Fox Memorial Lecture, The.—Some Considerations on the Cells and Liquids of the Body.—G. A. Buckmaster, 154.

MacManus, Miss E. E. P., Resignation of, 57.
Malignant Disease, The Surgery of.—Grey Turner, 141.
Medical Library of the University of Bristol, 62, 109, 148, 225, 281.
Medical Teaching in Bristol, Early.—F. R. Cross, 73.
Meetings of Societies—
Bristol Medico-Chirurgical Society, 59, 141, 223, 277.
Bristol and Bath Surgical Club, 60.
Murgoci, Helen B.—Six Cases of Cancer Arising in Tissues within the Duodenal Loop, 195.

Neild, Newman.—Two cases of Angina Pectoris following Sepsis, 263.

Obituary—

Sir Isambard Owen, 61.

Observations on Pellagra.—E. Barton White and G. Hadfield, 31.

Ophthalmic Neonatorum, The Prophylaxis of.—A. E. Iles., 125.

Owen, Sir Isambard.—Obituary, 57, 61.

Parker, G.—A Visit to the Near East, 173.
Pathology of Coronary Occlusion, The. —G. Hadfield, 257.
Pellagra, Observations of.—E. B. White and G. Hadfield, 31.
Postgraduate Medical Work, 275.
President in Canada, The, 274.
Presidential Address — Anæsthesia. — A. L. Flemming, 1.

Rheumatic Heart Disease, The Geographical Distribution of, 140.
Royal Commission on Lunacy and Mental Disorder, The, Recommendations of.—J. V. Blachford, 15.

Sheriff of Bristol. — Major J. A. Arrowsmith-Brown, 274.
Statham, R. S. S.—Some Clinical Aspects of Dysmenorrhœa, 187; Some Clinical Aspects of Sterility, 41.
Sterility, Some Clinical Aspects of.—R. S. S. Statham, 41.

Todd, A. T.—The Treatment of Cancer, 144.
Turner, Grey.—The Surgery of Malignant Disease, 141.

University College, Anniversary of, 58.

Vertigo, The Significance of.—E. Watson-Williams, 119.
Visit to the Near East, A.— G. Parker, 173.

Watson-Williams, E.—The Significance of Vertigo, 119.
White, E. B. and Hadfield, G.—Observations of Pellagra, 31.

J. W. ARROWSMITH LTD., BRISTOL

